# On the Capacity of Amplitude Modulated Soliton Communication over Long Haul Fibers

**DOI:** 10.3390/e22080899

**Published:** 2020-08-15

**Authors:** Yu Chen, Iman Tavakkolnia, Alex Alvarado, Majid Safari

**Affiliations:** 1 Institute for Digital Communication, School of Engineering, University of Edinburgh, Edinburgh EH9 3FD, UK; I.Tavakkolnia@ed.ac.uk; 2 Information and Communication Theory Lab, Signal Processing Systems Group, Department of Electrical Engineering, Eindhoven University of Technology, 5600 MB Eindhoven, The Netherlands; a.alvarado@tue.nl

**Keywords:** achievable information rate, channel capacity, nonlinear fiber optics, soliton communication, solitonic interaction, variance normalizing transform

## Abstract

The capacity limits of fiber-optic communication systems in the nonlinear regime are not yet well understood. In this paper, we study the capacity of amplitude modulated first-order soliton transmission, defined as the maximum of the so-called time-scaled mutual information. Such definition allows us to directly incorporate the dependence of soliton pulse width to its amplitude into capacity formulation. The commonly used memoryless channel model based on noncentral chi-squared distribution is initially considered. Applying a variance normalizing transform, this channel is approximated by a unit-variance additive white Gaussian noise (AWGN) model. Based on a numerical capacity analysis of the approximated AWGN channel, a general form of capacity-approaching input distributions is determined. These optimal distributions are discrete comprising a mass point at zero (off symbol) and a finite number of mass points almost uniformly distributed away from zero. Using this general form of input distributions, a novel closed-form approximation of the capacity is determined showing a good match to numerical results. Finally, mismatch capacity bounds are developed based on split-step simulations of the nonlinear Schr$\ddot{o}$dinger equation considering both single soliton and soliton sequence transmissions. This relaxes the initial assumption of memoryless channel to show the impact of both inter-soliton interaction and Gordon–Haus effects. Our results show that the inter-soliton interaction effect becomes increasingly significant at higher soliton amplitudes and would be the dominant impairment compared to the timing jitter induced by the Gordon–Haus effect.

## 1. Introduction

It is predicted that the capacity of data transfer network, mainly consists of optical fibers, will fall behind the data traffic demands in the near future [1]. The prediction implies the need for exploiting current optical fiber infrastructure to their limits before migrating to the next generation of optical fiber systems. However, the fundamental information transmission capacity of the most basic optical fiber link (i.e., standard single-mode fiber) is not fully known in the nonlinear regime. Different approaches have been used to tackle this problem in the literature including the recent application of nonlinear Fourier transform (NFT) to approach the limits of the nonlinear optical fiber [2,3]. Using NFT, the nonlinear dispersive fiber channel, defined by the nonlinear Schr$\ddot{o}$dinger equation (NLSE), is transformed to linear channels in nonlinear spectral domain, redefining the capacity problem formulation for nonlinear optical fibers.

By applying NFT, the available degrees of freedom in temporal domain are transformed to two types of spectra in the nonlinear spectral domain, namely the discrete and continuous spectra. Therefore, NFT is regarded as a base for development of new techniques of data transmission, and different communication system designs have been proposed using NFT [4,5,6,7,8,9,10,11,12,13]. The performance of such NFT-employed system for long-haul communication is investigated by simulation and experiment [14,15]. However, it has been observed that the noise behavior is not trivial in these systems [16,17,18], and the performance largely depends on the design. Moreover, the application of NFT in estimating the capacity of nonlinear optical fibers is not straightforward since the NFT and inverse NFT (INFT) must be performed numerically and are computationally complex [19,20].

An estimation of the capacity of the nonlinear optical fiber by only signaling on its continuous spectrum defined by NFT is provided in [21,22]. Achievable rates have been predicted, but it has been shown that due to the signal dependency of the noise, the capacity will be saturated at high power. Moreover, several works in the literature have been focused on estimating the achievable information rates (AIR) of the fiber when the discrete spectrum (i.e., soliton transmission) is used as the signal space. In [23], a capacity lower bound for amplitude modulated first-order soliton communication system is estimated using a half-Gaussian input distribution. In [24], an achievable rate is estimated taken into account the Gordon–Haus effect that leads to timing jitter at the receiver. In [18], AIR is estimated for a more complicated system that modulates both the eigenvalue and the norming constant in the discrete spectrum. Assuming a receiver capable of detecting variable pulse duration, in [25], the time-scaled mutual information (MI) is numerically optimized considering the memoryless channel model for soliton communication.

In this paper, we investigate the capacity of the optical fiber channel when only a single discrete spectrum point is encoded and the data is mapped on the imaginary part of the corresponding eigenvalue. This is essentially equivalent to the amplitude modulated soliton communication in [26]. As mentioned above, a number of capacity bounds for such channel has been derived previously [18,23,24], and AIR in bits per second were also discussed in [25]. However, some intrinsic limitations, such as dependence of bandwidth on soliton amplitude and the interaction between neighboring soliton pulses have been ignored. Compared to the state-of-art works in the literature (e.g., [23,25]), we investigate the effect of channel memory induced by solitonic interaction, which is mostly ignored in the literature. In order to incorporate the time-bandwith degrees of freedom into the capacity problem formulation, we study the maximization of time-scaled MI similar to [25] but by assuming a more practical communication system that uses a fixed symbol duration (i.e., soliton pulse width). A general form of capacity-approaching input distributions are proposed through the optimization of an approximated normalized channel model, providing important insights into the optimal design of soliton communication systems. In addition, an analytical estimation of the capacity of amplitude modulated soliton transmission is provided.

This paper is structured as follows: In Section 2, we initially consider a commonly used memoryless non-Gaussian channel model for the imaginary part of the eigenvalue [16]. By applying the variance normalizing transform (VNT) [22,27], the original channel is transformed into an equivalent channel with normalized noise power, which is then approximated by a unit-variance additive white Gaussian noise (AWGN) model in Section 3. Taking into account a peak amplitude constraint imposed by bandwidth limitations, the capacity in bits/normalized time and its corresponding input distribution are estimated using the proposed AWGN model and also an approximate analytical approach. Next, in the Section 4, we consider the effect of channel memory by developing the mismatch capacity bounds based on the split-step simulation of single soliton and soliton sequence transmissions over the NLSE. Based on the mismatch capacity results, the impact of inter-soliton interaction and Gordon–Haus effects on the capacity of soliton communication systems is studied.

## 2. Channel Model

At a low launch power, the optical fiber channel can be modeled as a linear dispersive channel impaired by AWGN noise. However, the Kerr nonlinearity becomes significant when the signal power increases to allow transmission over long haul fibers. The propagation of the complex envelope of a narrowband optical field in a standard single-mode fiber can be described by the stochastic nonlinear Schr$\ddot{o}$dinger equation (NLSE), as discussed in ([28], Chapter 4). Assuming the fiber loss to be perfectly compensated by an ideal distributed Raman amplification, the NLSE is given as

(1) $$\frac{\partial Q(T,Z)}{\partial Z}=-j\frac{\beta_{2}}{2}\frac{\partial^{2}Q\left(T,Z\right)}{\partial T^{2}}+j\gamma {\left|Q\left(T,Z\right)\right|}^{2}Q\left(T,Z\right)+N\left(T,Z\right),$$

where Q(T,Z) denotes the complex envelope of the optical field, N(T,Z) represents the amplifier spontaneous emission (ASE) noise term, *T* and *Z* are time and propagation distance, and $\beta_{2}$ and γ indicate group velocity dispersion and Kerr nonlinearity respectively. Note that the fiber loss term α here is omitted since ideal distributed Raman amplification is assumed. The ASE noise is modeled by a zero mean white Gaussian noise with autocorrelation $E\left[N\left(T,Z\right)N^{*}\left(T^{\prime },Z^{\prime }\right)\right]=N_{\mathrm{ASE}}\delta \left(T-T^{\prime }\right)\delta \left(Z-Z^{\prime }\right)$. The spectral density of the noise in [W/(km · Hz)] is $N_{\mathrm{ASE}}=\alpha h\nu_{0}K_{T}$ for the ideal distributed Raman amplification assumed in this work, where $h\nu_{0}$ denotes the photon energy and $K_{T}$ denotes the phonon occupancy factor. The NLSE could be normalized into the form

(2) $$j\frac{\partial q(t,z)}{\partial z}=\frac{\partial^{2}q\left(t,z\right)}{\partial t^{2}}+2{\left|q\left(t,z\right)\right|}^{2}q\left(t,z\right)+n\left(t,z\right),$$

with the corresponding normalized parameters as

(3) $$q=\sqrt{\gamma L_{D}}Q,\;z=Z/2L_{D},\;t=T/T_{0},$$

where dispersion length is defined as $L_{D}=T_{0}^{2}/\left|\beta_{2}\right|$, and normalizing time $T_{0}$ can be selected independent of other parameters. Consequently, the autocorrelation of the normalized noise is,

(4) $$E\left[n\left(t,z\right)n^{*}\left(t^{\prime },z^{\prime }\right)\right]=\sigma^{2}\delta \left(t-t^{\prime }\right)\delta \left(z-z^{\prime }\right),$$

where $\sigma^{2}=N_{\mathrm{ASE}}\frac{2\gamma L_{D}^{2}}{T_{0}}$ according to the normalization (3).

Using the inverse scattering method, NFT transforms the time domain optical signal into scattering data, consisting of continuous spectrum ρ(λ,z), eigenvalues ${\lambda_{m}\left(z\right)}_{m=1}^{M}$ and corresponding norming constants ${C_{m}\left(z\right)}_{m=1}^{M}$ which evolve linearly along the fiber in nonlinear spectral domain. It can be shown that, in a noise-free and interaction-free scenario, the eigenvalues $\lambda_{m}$ are preserved during the evolution along the fiber [29]. If only one eigenvalue exists at z=0 and ρ(λ,0)=0, the solution of NLSE is a first-order soliton, which can be described analytically as

(5) $$q\left(t,z\right)=2\eta e^{-2i\zeta t+4i\left(\zeta^{2}-\eta^{2}\right)z-i\left(\psi +\pi /2\right)}\mathrm{sech}\left(2\eta t-8\eta \zeta z-2\epsilon \right),$$

where the only eigenvalue is $\lambda_{1}=\zeta +i\eta$(η>0). Also, $e^{2\epsilon }=\frac{C_{1}}{2\eta }$ and $\psi =\arg\;C_{1}\left(z\right)$ where $C_{1}$ denotes the norming constant corresponding to eigenvalue $\lambda_{1}$.

The Energy of the soliton in (5) is equal to 4η, where the temporal width and bandwidth are proportional to 1/η and η respectively. Note that within this work, only the imaginary part of the eigenvalue is modulated and the real part is set to zero, i.e., η=A, ζ=0. Thus, at z=0, the input pulse can be expressed as

(6) q(t,z=0)=2Asech(2At).

The propagation of the soliton pulse over the fiber is described by NLSE, and at the receiver side, the eigenvalue can be detected by NFT or pulse energy estimation. If the detected eigenvalue is denoted as *R*, the channel model for this amplitude modulated first-order soliton transmission system can be described by a conditional PDF $P_{R|A}\left(r|a\right)$, which is non-Gaussian with a variance dependent on its mean [16,30]. Ignoring inter-soliton interactions, a memoryless channel model can be defined for the amplitude modulated soliton system based on a noncentral chi-squared distribution (NCX2) with 4 degrees of freedom as [16,23]

(7) $$P_{R|A}\left(r|a\right)=\frac{2}{\sigma_{N}^{2}}\sqrt{\frac{r}{a}}\exp\left(-\frac{2a+2r}{\sigma_{N}^{2}}\right)I_{1}\left(\frac{4\sqrt{ar}}{\sigma_{N}^{2}}\right),$$

where $I_{1}\left(\cdot \right)$ denotes the modified first order Bessel function of the first kind. The mean and variance of this distribution for large *a* are $\mu_{\mathrm{NCX}2}\left(a\right)=\sigma_{N}^{2}+a$ and $\sigma_{\mathrm{NCX}2}^{2}\left(a\right)=\frac{1}{2}\sigma_{N}^{4}+a\sigma_{N}^{2}$ respectively, where $\sigma_{N}^{2}=\frac{1}{2}\sigma^{2}\frac{L}{2L_{D}}$ at distance Z=L and $\sigma^{2}$ is the power spectral density of the normalized ASE noise as defined in Equation (4). It can be seen that the channel model (7) for the imaginary part of the eigenvalue (soliton amplitude, or soliton energy) is non-Gaussian with signal dependent variance. In the next section, we develop different approaches to estimate the capacity of the channel described by (7).

## 3. Capacity Formulation for Memoryless Soliton Communication Channel

Here, the capacity problem for the channel defined by the conditional PDF (7) is formulated considering a peak amplitude constraint since the bandwidth occupied by soliton pulses is directly related to their amplitudes. That is, the modulating data on higher amplitudes requires larger bandwidth while the maximum signal bandwidth is restricted by physical limitations. Moreover, in practical scenarios, peak power is also constrained due to device limitations. Another important issue that needs to be considered for soliton communications systems is that soliton pulses defined as in (6) are not time-limited, and thus, they should be truncated for practical implementations.

We define the practical width of a soliton pulse (denoted by $t_{s}$) as the temporal width that contains 1−δ of the soliton energy. Recalling the energy of the normalized soliton (6) is equal to 4A, this practical width can be obtained by solving the equation below for $t_{s}$

(8) $$\int_{-t_{s}/2}^{+t_{s}/2}|2A\mathrm{sech}\left(2At\right){|}^{2}dt=\left(1-\delta \right)4A,$$

which is given by

(9) $$t_{s}\left(A,\delta \right)=\frac{1}{2A}\ln\left(\frac{2}{\delta }-1\right),$$

where the fixed value δ should be sufficiently small to make the truncation error negligible compared to noise. For example, assuming that the soliton pulse width is defined based on containing 99.9% of its energy (δ=0.001), we have $t_{s}=3.8/A$. Noting that the temporal width of soliton pulses is inversely related to their amplitudes, we can also introduce a minimum amplitude constraint to limit the utilization of the temporal resources. Based on the constraints mentioned above, the capacity problem can be formulated as

(10) $$C_{\mathrm{bpcu}}=\underset{P_{A}\left(a\right):A\in \left\{0\right\}\cup \left[A_{\mathrm{lb}},A_{\mathrm{ub}}\right]}{\sup}I(A;R),$$

where $C_{\mathrm{bpcu}}$ denotes the capacity in bits per symbol per channel use, I(A;R) represents the MI. Denoting the transmitted and received eigenvalues with random variables *A* and *R* respectively, $A_{\mathrm{ub}}$ is the maximum amplitude constraint determined by maximum bandwidth or peak power and $A_{\mathrm{lb}}$ is the minimum amplitude constraint determined by the maximum allowed symbol duration. Note that we also consider the possibility of transmitting no soliton over a symbol duration (i.e., off symbol) with probability $p_{0}$, which is denoted by A=0 here.

Noting that the signal space and the temporal resources are inter-related in the underlying soliton communication system, we will use an alternative capacity formulation that maximizes time-scaled MI [25] to get better insights into AIRs of the system in bits per second. Unlike [25], we assume a fixed symbol duration for all transmitted solitons to facilitate practical implementation. Since the pulse width is inversely related to the amplitude of the soliton, the minimum nonzero soliton amplitude $A_{\min}\geq A_{\mathrm{lb}}$ (i.e., maximum pulse width) in a given input distribution determines the symbol duration. Note that $A_{\min}$ is not necessarily equal to the minimum amplitude constraint $A_{\mathrm{lb}}$ and $P\left(A<A_{\min}\right)=p_{0}$. The time-scaled MI (MI) is thus defined as

(11) $$R\left(A;R\right)=\frac{I(A;R)}{t_{s}\left(A_{\min},\delta \right)},$$

where MI is divided by the normalized symbol duration, resulting in a unit of [bits/normalized time]. The data rate in [bits/second] can be estimated by dividing the time-scale MI (11) with the normalizing time $T_{0}$ in (3). The corresponding time-scaled capacity formulation is then given by

(12) $$C=\underset{P_{A}\left(a\right):A\in \left\{0\right\}\cup \left[A_{\mathrm{lb}},A_{\mathrm{ub}}\right]}{\sup}R(A;R).$$

Note that the minimum amplitude constraint $A_{\mathrm{lb}}$ can be also relaxed, since it is already inherently imposed by the modified objective function, i.e., the time-scaled MI. This is because the optimal solution would not include the small soliton amplitudes that consume the available temporal resources inefficiently due to their very large pulse width. Hence the capacity problem can be also written as

(13) $$C=\underset{P_{A}\left(a\right):A\in \left[0,A_{\mathrm{ub}}\right]}{\sup}R(A;R).$$

In Section 3.2, it is shown that a minimum nonzero soliton amplitude $A_{\min}$ naturally appears in the optimal distribution of the capacity problem in (13).

### 3.1. Equivalent Channel Model Based on VNT

To simplify the capacity analysis, similar to the method used in [22,30,31,32], variance normalizing transform (VNT) is applied here to transform the original signal-dependent noise channel to a channel with a fixed noise power at sufficiently large signal-to-noise ratios. In general, the VNT can be applied to any random variable *R* where its variance $\sigma_{R}^{2}$ is related to the mean $\mu_{R}$ as $\sigma_{R}^{2}=f^{2}\left(\mu_{R}\right)$. Then the variance of the transformed random variable, Y=T(R), is normalized to one (i.e., mean independent) at sufficiently large values of $\mu_{R}$. The general form of VNT can be written based on [33] as

(14) $$T\left(u\right)=\int \frac{1}{f(u)}du.$$

Therefore the normalized random variable Y=T(R) has the moments $\sigma_{Y}^{2}\approx 1$ and $\mu_{Y}=E\left[y\right]\approx T\left(\mu_{R}\right)$ for sufficiently large value of $\mu_{R}$. Substituting the statistics of the NCX2 channel $\mu_{\mathrm{NCX}2}\left(a\right)=\sigma_{N}^{2}+a$ and $\sigma_{\mathrm{NCX}2}^{2}\left(a\right)=\frac{1}{2}\sigma_{N}^{4}+a\sigma_{N}^{2}=\sigma_{N}^{2}\left(\frac{1}{2}\sigma_{N}^{2}+a\right)=\sigma_{N}^{2}\left(\mu_{\mathrm{NCX}2}\left(a\right)-\frac{1}{2}\sigma_{N}^{2}\right)$ considered in this work, the VNT will be given as

(15) $$T\left(u\right)=\int \frac{1}{\sqrt{\sigma_{N}^{2}\left(u-\frac{1}{2}\sigma_{N}^{2}\right)}}du=2\sqrt{\frac{u}{\sigma_{N}^{2}}-\frac{1}{2}}\approx 2\sqrt{\frac{u}{\sigma_{N}^{2}}},$$

where the approximation is made for mathematical simplicity and due to the fact that the variance normalization itself defined by VNT is only precise at large values of $u/\sigma_{N}^{2}$ where the adopted approximation is also precise [22,27,31,32].

**Figure 1:**
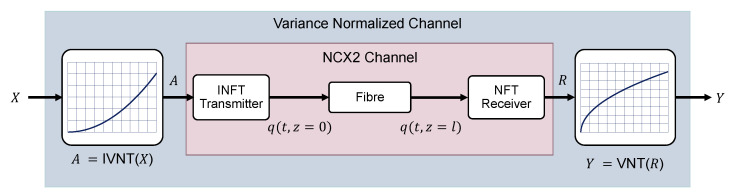
Block diagram of an amplitude modulated soliton communication system with inverse variance normalizing transform (IVNT) and VNT, *A* and *R* denote the transmitted and received soliton amplitude, *X* and *Y* denote the transformed input and output signals, and *q* denotes the time domain signal.

As shown in Figure 1, an equivalent soliton communication system can be defined based on the VNT approach where the noise power is signal-independent at large signal levels. Note that, in order to perform the coding and decoding at the same signal space, it is convenient to include both VNT and inverse VNT (IVNT) meaning that the soliton amplitude, *A*, is determined from the original input data X=T(A) as

(16) $$A=T^{-1}\left(X\right)=\sigma_{N}^{2}\frac{X^{2}}{4}.$$

Noting the square root form of the VNT defined in (15) and considering that the NCX2 model in (7) defines the channel between the soliton eigenvalues *A* and *R* in Figure 1, the equivalent channel model between the transformed random variables *X* and *Y* is described by a noncentral chi (NCX) conditional PDF as

(17) $$P_{Y|X}\left(y|x\right)=\frac{y^{2}}{x}\exp\left(-\frac{y^{2}+x^{2}}{2}\right)I_{1}\left(xy\right),$$

where $X=T\left(A\right)=2\sqrt{A}/\sigma_{N}$ and $Y=T\left(R\right)=2\sqrt{R}/\sigma_{N}$.

The capacity in bit per symbol of the system in (10) can then be rewritten based on the random variables *X* and *Y* as

(18) $$C_{\mathrm{bpcu}}=\underset{P_{X}\left(x\right):X\in \left\{0\right\}\cup \left[X_{\mathrm{lb}},X_{\mathrm{ub}}\right]}{\sup}I\left(X;Y\right),$$

where $X_{\mathrm{lb}}=T\left(A_{\mathrm{lb}}\right)$ and $X_{\mathrm{ub}}=T\left(A_{\mathrm{ub}}\right)$. Moreover, the corresponding time-scaled capacity formulation is given by

(19) $$C=\underset{P_{X}\left(x\right):X\in \left\{0\right\}\cup \left[X_{\mathrm{lb}},X_{\mathrm{ub}}\right]}{\sup}R(X;Y),$$

or based on the relaxed constraint as

(20) $$C=\underset{P_{X}\left(x\right):X\in \left[0,X_{\mathrm{ub}}\right]}{\sup}R(X;Y),$$

where the time-scaled MI can be written as

(21) $$R\left(X;Y\right)=\frac{I(X;Y)}{t_{s}\left(A_{\min},\delta \right)}=\frac{\sigma_{N}^{2}X_{\min}^{2}}{2\ln(2/\delta -1)}I\left(X;Y\right),$$

and $X_{\min}$ denotes the minimum nonzero symbol amplitude, i.e., $A_{\min}=T^{-1}\left(X_{\min}\right)=\sigma_{N}^{2}X_{\min}^{2}/4$. It is important to notice that the VNT transformation does not affect the MI between input and output, i.e., I(A;R)=I(X;Y), since the VNT function (15) is a monotonic and invertible function within the interested domain (See Lemma in [22]). Hence, the capacity formulations in (12) and (19) are equivalent.

### 3.2. Approximate AWGN Channel Model

It has been shown that the probability distribution of the normalized random variable after VNT tends to Gaussian distribution for a family of originally non-Gaussian probability distributions [22,31]. In this section, we first show that this is also true for the NCX distribution (17) in a Kullback–Leibler (KL) divergence sense. This inspires us to propose an approximate AWGN channel model to describe the amplitude modulated soliton communication system after VNT transformation as

(22) Y=X+Γ,

where the additive noise Γ is Gaussian with zero mean and unit variance.

**Proposition** **1.** *The KL divergence between the NCX distribution, $P_{Y|X}\left(y|x\right)$, given in (17) and a Gaussian distribution $Q_{Y|X}\left(y|x\right)$ with mean x and unit variance tends to zero for a sufficient large x, that is*

(23) $$\lim_{x\to +\infty }D_{\mathrm{KL}}\left(P,Q|x\right)=0,$$

*where KL divergence, $D_{\mathrm{KL}}\left(P,Q|x\right)$, is defined as*

(24) $$D_{\mathrm{KL}}\left(P,Q|x\right)=\int_{-\infty }^{+\infty }P_{Y|X}\left(y|x\right)\ln\frac{P_{Y|X}\left(y|x\right)}{Q_{Y|X}\left(y|x\right)}dy,$$

**Proof of Proposition** **1.** The detailed proof of Proposition 1 is shown in Appendix A. □

Proposition 1 indicates that the NCX channel model (17) behaves similar to the approximate AWGN channel for a sufficiently large *x*. For example, The KL divergence $D_{\mathrm{KL}}$ is estimated as small as $1.77\times 10^{-12}$ for x=86.67. This is by assuming that the pulse width contain 99.9% of the energy (δ=0.001) and some typical fiber parameters as in Table 1. Next, we will show that the proposed approximate AWGN channel converges to the original NCX channel at sufficiently large large $X_{\mathrm{lb}}$.

**Theorem** **1.** *Given the input $X\in \{0\cup \left[X_{\mathrm{lb}},X_{\mathrm{ub}}\right]\}$ at a sufficiently large $X_{\mathrm{lb}}$, the mismatch capacity of the NCX channel with the approximate AWGN channel defined by (22) as auxiliary channel converges to the actual capacity of the NCX channel.*

**Proof of Theorem** **1.** The detailed proof of Theorem 1 is shown in Appendix B. □

In [34,35], it is shown for the AWGN channel with amplitude constraints that the capacity-achieving distribution is discrete with a finite number of mass points for such channels. An upper bound is proposed in [36] for the number of mass points. However, these works focus on the MI-based capacity formulation. In the next Proposition, we extend the result in [34] to show the discreteness of the optimal solution to the time-scaled MI maximization problem for the proposed approximate AWGN channel.

**Proposition** **2.** *Given an AWGN channel with the input amplitude constraint of $X\in \{0\cup \left[X_{\mathrm{lb}},X_{\mathrm{ub}}\right]\}$ and $X_{\mathrm{lb}}\to \infty$, the optimal input distribution for the capacity formulation in (19) is discrete with a finite number of mass points.*

**Proof of Proposition** **2.** The detailed proof of Proposition 2 is shown in Appendix C. □

Now, approximating the channel in (19) with an AWGN model based on Theorem 1 and considering the conclusion of Proposition 2 on the discreteness of the optimal input distribution asymptotically, the MI between *X* and *Y* can be expressed as

(25) $$\begin{array}{cc} I(X;Y) & =h(Y)-h(Y|X)=h(Y)-h(\Gamma )\\ \; & =\sum_{k=0}^{M}\int p_{X}\left(x_{k}\right)Q_{Y|X}\left(y|x_{k}\right)\log_{2}\frac{1}{\sum_{j=0}^{M}p_{X}\left(x_{j}\right)Q_{Y|X}\left(y|x_{j}\right)}dy-\log_{2}\sqrt{2\pi e}, \end{array}$$

where h(Y) denotes the output differential entropy, h(Γ) denotes the differential entropy of the unit variance AWGN noise, $x_{k}$ and $p_{X}\left(x_{k}\right)$ denote the input symbols and their corresponding probabilities within the input source alphabet, *M* denote the size of the nonzero alphabet, $x_{0}=0$ and $p_{X}\left(x_{0}\right)=p_{0}$ denotes the corresponding probability. Hence, the problem in (19) can be rewritten as

(26) $$C=\max_{M}\left[\max_{\left[x,p_{X}\right]:\;x_{k}\in \left\{0\right\}\cup \left[X_{\mathrm{lb}},X_{\mathrm{ub}}\right]}R\left(X;Y\right)\right],$$

where the time-scaled MI function R(X;Y) is a function of two (M+1)-length vectors x and $p_{X}$ which denote the mass points and their probabilities. As mentioned in the previous sections, the minimum amplitude constraint can be also relaxed yielding

(27) $$C=\max_{M}\left[\max_{\left[x,p_{X}\right]:\;x_{k}\in \left[0,X_{\mathrm{ub}}\right]}R\left(X;Y\right)\right].$$

Since the input distribution is discrete, the vector $\left[x,p_{X}\right]$ is sufficient to describe the input random variable *X*. The discreteness of the capacity-achieving input distribution allows for numerical evaluation of the capacity expression using similar algorithms as in [30,34]. In this work, the optimization over $\left[x,p_{X}\right]$ is performed using an interior-point optimizer in MATLAB given the number of nonzero mass point is fixed at *M*. The optimization on *M* is then performed based on an exhaustive search approach which will keep increasing *M* until additional mass points can no longer improve the optimized time-scaled MI.

**Figure 2:**
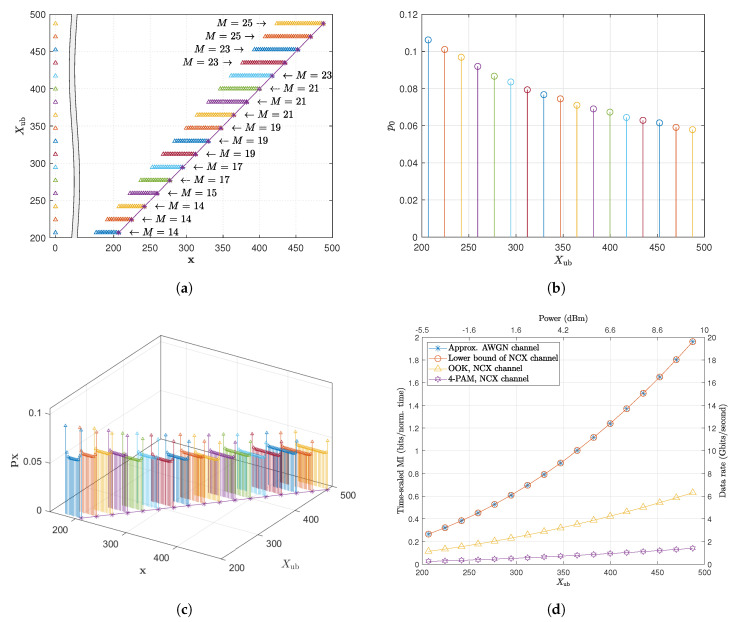
The optimal input distribution and the corresponding optimized time-scaled mutual information (MI) obtained as the numerical solution of (26) subject to the peak amplitude constraint X_ub_ assuming *δ* = 0.001. (**a**) The location of the optimal mass points (the peak amplitude is shown as the purple solid line with star) (**b**) The optimal probability of the mass point at zero (i.e., off symbol) (**c**) The optimal probabilities of the nonzero mass points, (**d**) The maximum Time-scaled MI given based on the solution of (26) and the lower bounds on the time-scaled capacity of the original noncentral chi-squared distribution (NCX) channel achieved by using different input distributions, including, on-off keying (OOK), 4 pulse amplitude modulation (4-PAM) and the input distribution given in (**a**) to (**c**). Note that the additional power axis denotes the power level of the solitons corresponds to the peak amplitude X_ub_ assuming *δ* = 0.001.

Figure 2 shows the capacity-achieving distributions obtained by solving (26) and the corresponding capacity estimation using the optimized input distribution. For these results, we assume an ideal distributed Raman amplified 2000 km fiber with the parameters detailed in Table 1. Using the constraint from $X_{\mathrm{ub}}=200$ to $X_{\mathrm{ub}}=500$. This range of peak amplitude constraint corresponds to the range of maximum eigenvalue from $A_{\mathrm{ub}}=0.4$ to $A_{\mathrm{ub}}=2.5$, which represent the peak optical power −5 dBm and +10 dBm, respectively.

In Figure 2a–c, the optimal distributions are shown for various peak amplitude constraints $X_{\mathrm{ub}}$. The figures show that the optimal distributions consist of an isolated mass point at zero (off symbol), and a uniform-like distribution starting from a minimum nonzero symbol (denoted by $X_{\min}$) to the maximum symbol amplitude (denoted by $X_{\max}=X_{\mathrm{ub}}$). It is also important to point out that the probabilities at $X_{\min}$ and $X_{\max}$ getting closer to the probabilities of the mass points in between as $X_{\mathrm{ub}}$ increases, showing a convergence towards a uniform distribution. Note that the results in [25] shows a nonuniform distribution of optimal mass points since the pulse width is assumed to be variable.

Figure 2d presents the capacity of the approximate AWGN channel based on the solution of (26) as well as some lower bounds on the capacity of the original NCX channel (17). The best lower bound is obtained by applying the optimal distribution of the approximate AWGN channel as in Figure 2a–c to the time-scaled MI of the NCX channel. This lower bound precisely overlaps with the capacity of the approximate AWGN channel, further confirming the result of Theorem 1, in a MI sense, i.e., that the AWGN channel is a very good approximation of the NCX channel within the range of consideration. Figure 2d also includes the time-scaled MI estimated for the transmission of conventional on-off keying (OOK) and 4 pulse amplitude modulation (4-PAM) signals over the original NCX channel. As expected, both conventional modulations show lower time-scaled MI comparing to the optimized input distribution. However, the conventional 4-PAM signal achieves even lower time-scaled MI than OOK. This is due to the fact that the fixed symbol duration is inversely related to the amplitude of the minimum nonzero amplitude $X_{\min}$, which is $X_{\min}=X_{\mathrm{ub}}/3$ for 4-PAM but $X_{\min}=X_{\mathrm{ub}}$ for OOK. In general, for a *K*-PAM modulation scheme, the time-scaled MI can be upper bounded by the time-scaled source entropy, $\frac{H(X)}{t_{s}\left(A_{\min},\delta \right)}=\frac{\sigma_{N}^{2}X_{\min}^{2}}{2\ln(2/\delta -1)}\log_{2}\left(K\right)$, where the $X_{\min}=X_{\mathrm{ub}}/\left(K-1\right)$. It can be then shown that the time-scaled source entropy for *K*-PAM will always decrease with respect to *K* for K≥2. This suggests that *K*-PAM with higher *K* cannot achieve better time-scaled MI than OOK. It is also worth noting that some of the sub-optimal distributions proposed in the literature (e.g., the half-Gaussian bound proposed in [23]) is not included here as the half-Gaussian input source would give a zero time-scaled MI when a fixed symbol duration is considered as in this paper.

### 3.3. Analytical Capacity Approximation

Inspired by the optimal input distributions obtained in the last section as presented in Figure 2, in this section, we focus on developing an analytical approach for time-scaled capacity estimation of the soliton communication system. Assuming that the peak amplitude constraint $X_{\mathrm{ub}}$ is sufficiently large, Figure 2 shows that the capacity-achieving input distribution obtained by solving (26) is discrete with a finite number of mass points including an almost uniform distribution within $\left[X_{\min},X_{\max}=X_{\mathrm{ub}}\right]$, and an additional mass points at zero, where the optimal $X_{\min}$ needs to be found by solving the optimization problem. We therefore consider a general form of discrete input distribution with a mass point at zero with probability $p_{0}$ and a discrete uniform distribution within $\left[X_{\min},X_{\max}\right]$ to find an analytical estimation of the solution to the capacity problem given in (26). Note that the upper boundaries of the distribution is denoted by $X_{\max}\leq X_{\mathrm{ub}}$ rather than $X_{\max}=X_{\mathrm{ub}}$ to keep it inline with the peak amplitude constraint introduced earlier.

To write the corresponding MI based on (25), we first need to define the statistics of the channel output given the input signal parameters, $P_{Y}\left(y|p_{0},X_{\min},X_{\max}\right)$. In order to make the capacity analysis tractable, we make an approximation that the distribution of the noisy output signal *Y* given the transmission of nonzero mass points, i.e., $P_{Y}\left(y|X\in \left[X_{\min},X_{\max}\right]\right)$ is approximated by a continuous uniform distribution within the range $\left[X_{\min},X_{\max}\right]$. This approximation is reasonable when the number of mass points *M* are large and the noise variance is small compared to the signal level. Based on this approximation and also considering the Gaussian noise added to the zero mass point, we can write

(28) $$P_{Y}\left(y|p_{0},X_{\min},X_{\max}\right)\approx p_{0}f_{G}\left(y\right)+\frac{1-p_{0}}{X_{\max}-X_{\min}}u\left(y|X_{\min},X_{\max}\right),$$

where the $f_{G}\left(\cdot \right)$ denotes the PDF of a zero mean, unit variance Gaussian distribution and $u(y|X_{\min},X_{\max})$ denotes the step function that is equal to 1 when *y* is within $\left[X_{\min},X_{\max}\right]$ and 0 otherwise.

Considering the approximate PDF in (28), we now calculate the differential entropy of the received signal as

(29) $$\begin{array}{cc} h(Y) & =\int_{-\infty }^{+\infty }P_{Y}\left(y\right)\log_{2}\frac{1}{P_{Y}}dy\\ \; & \overset{a}{\approx }\int_{-\infty }^{X_{\min}}p_{0}f_{G}\left(y\right)\log_{2}\frac{1}{P_{Y}}dy+\int_{X_{\min}}^{+\infty }P_{Y}\left(y\right)\log_{2}\frac{1}{P_{Y}}dy\\ \; & \overset{b}{\approx }\int_{-\infty }^{X_{\min}}p_{0}f_{G}\left(y\right)\log_{2}\frac{1}{p_{0}f_{G}\left(y\right)}dy+\int_{X_{\min}}^{X_{\max}}\frac{1-p_{0}}{X_{\max}-X_{\min}}\log_{2}\frac{X_{\max}-X_{\min}}{1-p_{0}}dy\\ \; & =p_{0}\log_{2}\frac{1}{p_{0}}+p_{0}\log_{2}\sqrt{2\pi e}+\left(1-p_{0}\right)\log_{2}\frac{X_{\max}-X_{\min}}{1-p_{0}}, \end{array}$$

where the approximation *a* leads from applying the approximate output distribution in (28), and the approximation *b* is valid under the assumption that $X_{\min}\gg 0$, i.e., $f_{G}\left(y\geq X_{\min}\right)\approx 0$. Substituting (29) into the Equation (25), the approximated MI is then given as a function of $p_{0}$, $X_{\min}$ and $X_{\max}$ as

(30) $$I_{\mathrm{app}}\left(X;Y\right)=p_{0}\log_{2}\frac{1}{p_{0}}+\left(1-p_{0}\right)\log_{2}\frac{X_{\max}-X_{\min}}{1-p_{0}}-\left(1-p_{0}\right)\log_{2}\sqrt{2\pi e}.$$

Noting that the scaling time (9) is a function of the minimum mass point $X_{\min}$, the approximate time-scaled MI function $R_{\mathrm{app}}\left(X;Y\right)$ is then given as

(31) $$R_{\mathrm{app}}\left(X;Y\right)=\frac{\sigma_{N}^{2}X_{\min}^{2}}{2\ln(2/\delta -1)}\left[p_{0}\log_{2}\frac{1}{p_{0}}+\left(1-p_{0}\right)\log_{2}\frac{X_{\max}-X_{\min}}{1-p_{0}}-\left(1-p_{0}\right)\log_{2}\left(\sqrt{2\pi e}\right)\right].$$

**Theorem** **2.** *Given the approximated time-scaled MI function in (31), the solution to the capacity problem given in (26), is obtained as*

(32) $$C_{\mathrm{app}}=R_{\mathrm{app}}{\left(X;Y\right)|}_{p_{0}^{*},X_{\min}^{*},X_{\max}^{*}},$$

*where the optimal parameters of the input distribution are given as*

(33) $$\begin{array}{cc} X_{\max}^{*} & =X_{\mathrm{ub}}, \end{array}$$

(34) $$\begin{array}{cc} X_{\min}^{*} & =\left(X_{\mathrm{ub}}+\sqrt{2\pi e}\right)\left[1-\frac{1}{2W(\frac{X_{\mathrm{ub}}}{2\sqrt{2\pi }}+\frac{\sqrt{e}}{2})}\right], \end{array}$$

(35) $$\begin{array}{cc} p_{0}^{*} & =\frac{2\sqrt{2\pi e}W\left(\frac{X_{\mathrm{ub}}}{2\sqrt{2\pi }}+\frac{\sqrt{e}}{2}\right)}{X_{\mathrm{ub}}+\sqrt{2\pi e}}, \end{array}$$

*where W(·) denotes the Lambert W function.*

**Proof of Theorem** **2.** The detailed proof of Theorem 2 is shown in Appendix D. □

**Figure 3:**
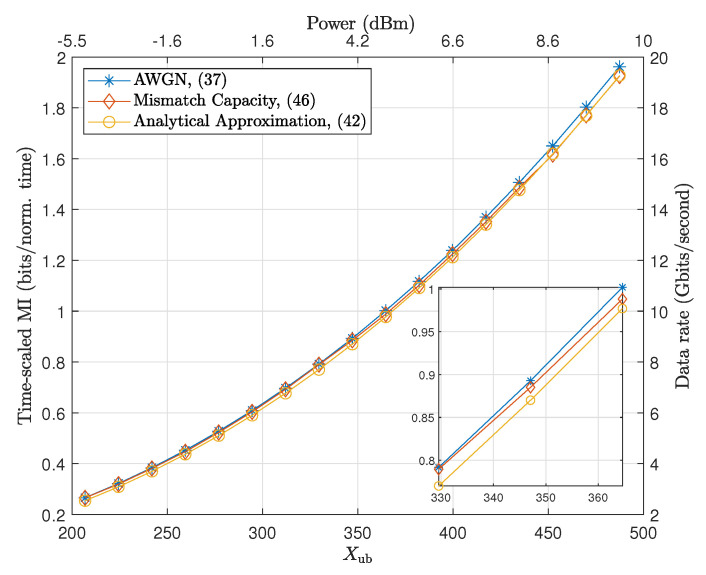
Time-scaled MI estimated from the additive white Gaussian noise (AWGN) model optimization in (26), the analytical approximation in (32), and the corresponding mismatch capacity bound in (36) for a 2000 km long fiber, assuming δ=0.001. The subplot shows the zoomed figure of $X_{\mathrm{ub}}\in \left[330,380\right]$.

Using Theorem 2, the approximate solution to the capacity problem in (26) can be calculated analytically. As it can be observed in Figure 3, this approximate capacity result demonstrates a close match to the exact capacity results obtained numerically.

## 4. Mismatch Capacity for Soliton Communication over the NLSE Channel

So far, we have focused on the capacity estimation of the first-order soliton transmission based on the commonly used memoryless channel model defined by the noncentral chi-squared distribution in (7). In this section, we study the capacity limits of the soliton transmission over a more realistic description of the fibre-optic channel defined by the NLSE. Hence, both the Gordon–Haus effect and the nonlinear interactions between adjacent soliton pulses can be incorporated into the capacity analysis. For this purpose, we use the numerical evaluation of mismatch capacity bounds based on split-step simulation of the NLSE. The mismatch capacity approach is commonly used to provide a lower bound on the capacity of a communication system, by assuming a mismatch distribution for decoding the received signal [32,37]. If the mismatch distribution is denoted by $Q_{Y|X}\left(y|x\right)$ and the real channel statistics is denoted by $P_{Y|X}\left(y|x\right)$, the time-scaled mismatch capacity bound for a discrete input signal is expressed as

(36) $$\begin{array}{cc} C_{\mathrm{Mismatch}} & =\frac{1}{t_{s}\left(A_{\min},\delta \right)}\sum_{k=0}^{M}\int_{-\infty }^{+\infty }p_{X}\left(x_{k}\right)P_{Y|X}\left(y|x_{k}\right)\log\frac{Q_{Y|X}\left(y|x_{k}\right)}{\sum_{j=0}^{M}p_{X}\left(x_{j}\right)Q_{Y|X}\left(y|x_{j}\right)}dy\\ \; & =\frac{1}{t_{s}\left(A_{\min},\delta \right)}\sum_{k=0}^{M}p_{X}\left(x_{k}\right)E_{P_{Y|X}\left(y|x_{k}\right)}\left[\log\frac{Q_{Y|X}\left(y|x_{k}\right)}{\sum_{j=0}^{M}p_{X}\left(x_{j}\right)Q_{Y|X}\left(y|x_{j}\right)}\right], \end{array}$$

where $p_{X}\left(x_{j}\right)$ denotes the input probability of symbol $x_{j}$ taken from optimization (26), and $E_{P_{Y|X}\left(y|x_{k}\right)}\left[\cdot \right]$ denotes an expectation operation over the channel model $P_{Y|X}\left(y|x_{k}\right)$. Recall from Section 3.1 that the unit-variance Gaussian distribution and the NCX distribution are well matched for the interested range of interest. Thus, a unit-variance Gaussian distribution $Q_{Y|X}\left(y|x\right)$ is a reasonable mismatch distribution to be employed in the calculation of the mismatch capacity.

To take into account the impairments introduced by ASE noise, such as Gordon–Haus timing jitter, as well as intersoliton interaction effects, we use the split-step method to simulate the propagation of single soliton or soliton sequence transmission over the fiber. Hence many realizations of the fiber-optic channel can be generated based on the simulation of NLSE to establish the statistics of the realistic channel given the capacity-approaching input distribution obtained in Section 3.2, (i.e., $P(y|x_{k})$). The generated channel statistics can then be used to numerically estimate the mismatch capacity in (36) through a Monte Carlo approach. Noting that the input distribution applied here is not necessarily the optimal distribution for the realistic channel, our results, $C_{\mathrm{Mismatch}}$, provide a lower bound on the mismatch capacity, which in turn gives a lower bound on the capacity of the realistic soliton communication system. The simulation of the channel realization required for the Monte Carlo estimation of mismatch capacity is generated following each function block of the proposed system as in Figure 1. The pulses correspond to the input alphabet will be transmitted into a simulated fiber perturbed by ASE noise via split step Fourier method based on NLSE (1). The output pulse from the simulated fiber will then be put through an NFT detector, which extracts the eigenvalue *R* from the detected pulse. The received eigenvalue *R* will then be VNT transformed into the transformed domain for decoding the information. Unless otherwise mentioned, δ=0.001 is assumed to calculate the soliton duration, i.e., 99.9% soliton energy pulse-width.

### 4.1. Mismatch Capacity for Single Soliton Transmission

We first focus on single soliton transmission over the NLSE which takes into account the Gordon–Haus effect while ignoring the inter-soliton interaction effects. Using identical fiber parameters as in Table 1, Figure 3 compares the time-scaled mismatch capacity calculated based on 1000 realizations per possible symbol for $X_{\mathrm{ub}}\in \left[200,500\right]$ with the time-scaled capacity of AWGN model obtained in Section 3.2 and the analytical approximation derived in Section 3.3. From Figure 3, it can be observed that the time-scaled MI increases as the peak amplitude constraint increases. It is also observed all the curves provide a well-matched estimations of the capacity, confirming that the Gordon–Haus effect is not so significant within the range of interest here. Nevertheless, we can see that, for larger $X_{\mathrm{ub}}$, the gap between mismatch and AWGN curves increases, which can be due to the stronger Gordon–Haus effect, that will be experienced by larger amplitude soliton pulses. Note that the timing jitter introduced by the Gordon–Haus effect can shift the soliton beyond the limited timing window over which the NFT is applied, which leads to energy loss and possible errors in eigenvalue detection.

### 4.2. Mismatch Capacity for Soliton Sequence Transmission

The memoryless channel model of soliton communication considered in Section 3 and in most of the literature is only valid when there is no intersoliton interactions, limiting the accuracy of the model to the cases where the sequence of soliton pulses are well separated. In this section, we use the mismatch capacity approach introduced above to provide some insights on the impact of inter-soliton interaction effects on the capacity of soliton communication systems. In the previous section, the performance of the system is discussed based on simulating the transmission of a single soliton pulse through a long haul fiber-optic channel, which neglects the inter-solitonic interactions. In this section, the transmission of a sequence of three soliton pulses is considered, where the middle soliton is considered to be the target soliton for detection. Meanwhile, the neighboring solitons (i.e., the first and the third solitons) are assumed to be independently and randomly selected based on the statistics of the input signal distribution taken from the solution of the AWGN capacity formulation in (26). Note that the pulse width of a soliton is a function of δ and $X_{\min}$ in the input signal distribution. The simulation is performed based on the same split step Fourier method employed in Section 4.1, while the NFT-based detection is only performed over the pulse width of the middle soliton.

It has been shown in [38] that, even in the absence of any noise, solitons can exert attracting or repelling forces on each other when they are not place far enough, and this leads to inter-soliton interaction effects. Thus, before implementing the soliton sequence transmission in the presence of the ASE noise, we intend to estimate the mean squared error (MSE) induced by the noiseless inter-soliton interaction to evaluate the significance of this effect for different soliton separations. Recall that the ASE noise power after VNT is normalized to 1. Hence, the inter-soliton interaction effect would be negligible relative to noise, if the inter-soliton interaction MSE is much less than 1, i.e.,

(37) $$MSE=E[{\left(Y_{\mathrm{nl}}-X\right)}^{2}]\ll 1,$$

where E[·] denotes expectation over all possible combination of the three-soliton sequences, $Y_{\mathrm{nl}}$ denotes the received VNT transformed eigenvalue in a noiseless scenario. The noiseless simulation is based on the identical simulation parameters as in Table 1 but in the absence of ASE noise (i.e., assuming noiseless ideal distributed Raman amplification) and using the input soliton amplitudes taken from the capacity-approaching distribution given in Section 3.2. In this section, the signaling of the solitons are based on four different δ parameters and their corresponding pulse width. Note that a smaller δ leads to a longer symbol duration as defined by (9), which results in more separation between solitons an thus less inter-soliton interaction.

**Figure 4:**
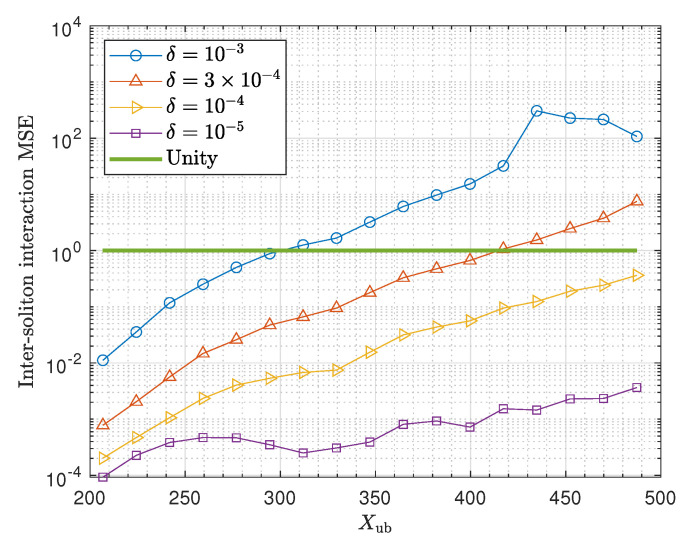
Inter-soliton interaction mean squared error (MSE) for different soliton pulse width determined by different values of δ and based on the link parameters stated in Table 1.

Figure 4 shows the inter-soliton interaction MSE estimated by simulating the transmission of all possible three-soliton sequences following the input distribution given in Section 3.2 assuming different values of δ. The overall trend of the MSE is increasing as the peak amplitude constraint $X_{\mathrm{ub}}$ is increasing. Moreover, as expected, decreasing the δ parameter reduces the MSE. In fact, reducing δ corresponds to the decreasing the fraction of energy truncation that essentially extends the soliton temporal separation. The additional temporal separation will reduce the force between the solitons [38], thus, the inter-soliton interaction is mitigated. Note that, for $\delta =10^{-3}$, the MSE goes beyond unity for $X_{\mathrm{ub}}>300$ as shown in Figure 4, meaning that the inter-soliton interaction effect becomes comparable to noise beyond that point, hence, the δ parameter needs to be reduced to maintain a low interaction effect. Similarly, it is observed that the MSE becomes comparable to noise for $\delta =3\times 10^{-4}$ beyond $X_{\mathrm{ub}}=400$.

**Figure 5:**
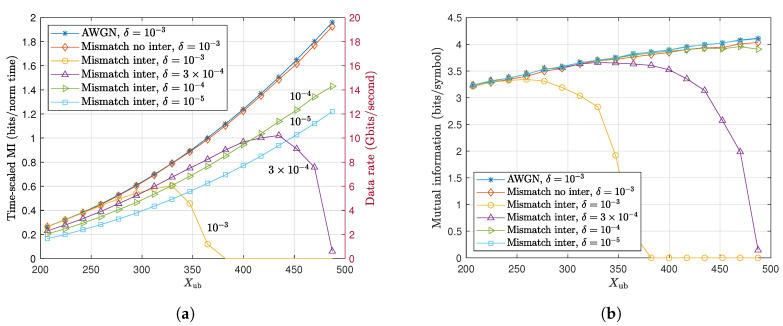
The capacity estimation of the soliton communication based on the AWGN model optimization in (26), and the mismatch capacity bounds in the presence (mismatch inter) or absence (mismatch no inter) of inter-soliton interaction effects in terms of (**a**) time-scaled MI and (**b**) MI, for different values of *δ* and the link parameters stated in Table 1.

In order to evaluate the impact of intersoliton interaction effect on the capacity of the system, Figure 5 shows the time-scaled capacity results and the corresponding MI calculated based on different proposed methods including the AWGN model and mismatch decoding with or without inter-soliton interaction effects for different values of δ. Figure 5a shows the significant impact of intersoliton interaction effects on the time-scaled capacity at higher peak amplitudes. For example, for $\delta =10^{-3}$, the time-scaled MI gradually drops beyond $X_{\mathrm{ub}}=300$ and tends to zero before $X_{\mathrm{ub}}=400$. It is also observed that when δ decreases, the longer symbol duration scales down the time-scaled MI in the whole range of $X_{\mathrm{ub}}$ but the efficiency of the communication system in combating intersoliton interaction effects improves (i.e., capacity drop shifts to higher soliton amplitudes). This indicates that there is a trade-off in selecting the parameter δ. On the one hand, a smaller δ mitigates more effectively both inter-soliton interaction and Gordon–Haus effects, and on the other hand, it reduces how efficiently the temporal resources are being used. Hence, in future work, δ also needs to be included in the capacity problem formulation. Nevertheless, Figure 5a gives an estimation of sensitivity of the time-scaled capacity with respect to δ by providing the mismatch results at different values of this parameter. Therefore, by taking the supremum of the curves with different δ values in different parts of the dynamic range, we can obtain a good estimation of the capacity lower bound in the presence of soliton interaction. For example, based on the available results, the capacity result at $\delta =10^{-3}$ is best up to $X_{\mathrm{ub}}=300$ while the capacity results for $\delta =3\times 10^{-4}$ and $\delta =10^{-4}$ are best in ranges $X_{\mathrm{ub}}\in \left[300,400\right]$ and $X_{\mathrm{ub}}>400$, respectively.

The MI results presented in Figure 5b is produced from scaling back the optimized time-scaled MI results in Figure 5a. It therefore focuses on how efficiently each soliton is decoded rather than how efficiently the temporal resources are being used. The figure shows that, for $\delta =10^{-3}$, the inter-soliton interaction effect strongly degrades the mismatch capacity beyond $X_{\mathrm{ub}}=300$ as expected from Figure 4 and Figure 5a. By reducing δ, it is observed that the inter-soliton interaction effect decreases and it almost matches the mismatch capacity results with no interaction at $\delta =10^{-4}$. This is also expected from Figure 4, as $\delta =10^{-4}$ shows MSE≪1 for most of the range of interest. In addition, the mismatch capacity at $\delta =10^{-5}$ even outperforms the mismatch capacity with no interaction and almost matches the AWGN result. This is because the mismatch with interaction at $\delta =10^{-5}$ corresponds to the transmission of a soliton sequence with longer symbol duration. The longer duration essentially eliminates both the Gordon–Haus effect as well as the interaction effects while this is not the case in the mismatch results with no interaction where we still assume shorter pulse width with $\delta =10^{-3}$. This also verifies the accuracy of the proposed AWGN approximation model compared to the realistic simulated channel when both the Gordon–Haus and inter-soliton interaction effects are negligible.

## 5. Conclusions

In this paper, we proposed a number of new approaches for estimating the capacity of the amplitude-modulated soliton communication systems. We provided insights into the AIRs of such systems when effects such as Gordon–Haus and inter-soliton interaction are present. The non-central Chi squared channel model that is commonly used in the literature was initially considered and was then approximated by a unit-variance AWGN channel by applying VNT. Using the approximated channel model and subject to a peak amplitude constraint, optimal input distribution and the corresponding capacity were obtained numerically. The optimized distributions are discrete with a mass point at zero corresponding to no soliton transmission as well as an almost uniform distribution of mass points spread in a range away from zero up to the peak amplitude constraint. Using this general form of the optimal distribution based on the approximate AWGN model and applying some mathematical simplifications, we developed an analytical expression to estimate the capacity of the soliton communication system. Despite the additional approximations, the analytical approach provides a close match to the results obtained numerically based on the AWGN model. The optimal input distribution based on AWGN model were also used to calculate the mismatch capacity of the soliton communication system using the split-step simulation of the realistic channel defined by the NLSE. The results show that the effect of inter-soliton interaction caused by limiting the soliton pulse width is stronger than the Gordon–Haus effect for long haul fibers operating in a range of launch powers up to 10 dBm. They also show the trade-off between extending the pulse width to avoid inter-soliton interaction and compressing the pulse width to improve the temporal efficiency.

In future works, the soliton pulse truncation factor δ can be included in the capacity problem formulation as an additional variable. This allows for a more comprehensive analysis of the soliton interaction effects. Moreover, the capacity problem based on the assumption of variable pulse width can be considered in the presence of soliton interaction effects. Another interesting problem related to this work is the capacity analysis of higher-order soliton transmissions.

## Figures and Tables

**Table 1:** Fiber Parameter.

length *L*	2000 km
Loss α	0.2 dB/km
Group velocity dispersion factor $\beta_{2}$	−2.1 × 10${}^{-26}$ $s^{2}/m$
Kerr nonlinearity factor γ	1.27 × 10${}^{-3}$ /W/m
Phonon occupancy $K_{T}$	1.13
Signal wavelength $\nu_{0}$	1.55 μm
Normalizing time $T_{0}$	0.1 ns

## Notations

*j*: $\sqrt{-1}$; Euler’s number: *e*; Absolute value: |·|; Expectation: E[·]; Modified first order Bessel function of the first kind: $I_{1}\left(\cdot \right)$; Probability density function (PDF) of *y*: $P_{Y}\left(y\right)$; Conditional PDF of *y* given *x*: $P_{Y|X}\left(y|x\right)$; MI between input *X* and output *Y*: I(X;Y); Time-scaled MI between input *X* and output *Y*: R(X;Y) [25]; KL divergence between distribution *P* and distribution *Q* given parameter *x*: $D_{\mathrm{KL}}\left(P,Q|x\right)$; Lower constraint: ${\left(\cdot \right)}_{\mathrm{lb}}$; Upper constraint: ${\left(\cdot \right)}_{\mathrm{ub}}$; Minimum nonzero value: ${\left(\cdot \right)}_{\min}$; Maximum nonzero value: ${\left(\cdot \right)}_{\max}$; Optimal value: ${\left(\cdot \right)}^{*}$; Lambert W function: W(·).

## Appendix A

**Proof of Proposition** **1.** Following a similar method as in [32], a non-negative term KL divergence is employed to evaluate difference between two distributions,

(A1) $$D_{\mathrm{KL}}\left(P,Q|x\right)=\int_{-\infty }^{+\infty }P_{Y|X}\left(y|x\right)\ln\frac{P_{Y|X}\left(y|x\right)}{Q_{Y|X}\left(y|x\right)}dy,$$

where *P* and *Q* denote the distributions, *x* indicates the given parameter(s) of the two distribution *P* and *Q*. Within this proof, $P_{Y|X}\left(y|x\right)$ is considered to be a noncentral chi distribution as

(A2) $$P_{Y|X}\left(y|x\right)=\frac{y^{2}}{x}\exp\left(-\frac{y^{2}+x^{2}}{2}\right)I_{1}\left(xy\right),$$

where $I_{1}\left(\cdot \right)$ denotes the modified Bessel function of the first kind, and the mean and variance of $P_{Y|X}$ are denoted with $\mu_{NCX}\left(x\right)$ and $\sigma_{\mathrm{NCX}}^{2}\left(x\right)$ respectively. $Q_{Y|X}\left(y|x\right)$ is considered as a Gaussian distribution with identical mean $\mu_{NCX}\left(x\right)$ and variance $\sigma_{\mathrm{NCX}}^{2}\left(x\right)$, i.e.,

(A3) $$Q_{Y|X}\left(y|x\right)=\frac{1}{\sqrt{2\pi \sigma_{NCX}^{2}\left(x\right)}}\exp\left(-\frac{{\left(y-\mu_{NCX}\left(x\right)\right)}^{2}}{2\sigma_{\mathrm{NCX}}^{2}\left(x\right)}\right).$$

To prove the convergence of the NCX distribution to a Gaussian distribution with mean *x* and unit variance at a sufficiently large *x*, we first verify the convergence of the moments of of the NCX distribution and then show its tendency to Gaussian distribution at large *x*. Taking the limits of the first and second moments at large values of *x*, we obtain

(A4) $$\lim_{x\to \infty }\mu_{\mathrm{NCX}}\left(x\right)=\lim_{x\to \infty }\sqrt{4+x^{2}-\sigma_{\mathrm{NCX}}^{2}\left(x\right)}=\lim_{x\to \infty }\sqrt{3+x^{2}}=x,$$

(A5) $$\lim_{x\to \infty }\sigma_{\mathrm{NCX}}^{2}\left(x\right)=\lim_{x\to \infty }4+x^{2}-\mu_{\mathrm{NCX}}^{2}\left(x\right)=\lim_{x\to \infty }4+x^{2}-\frac{\pi }{2}{\left[L_{1/2}^{\left(1\right)}\left(-\frac{x^{2}}{2}\right)\right]}^{2}=1,$$

which verifies the convergence of moments to the corresponding values in the theorem statement. Now substituting the NCX distribution (A2) and its corresponding Gaussian distribution (A3) into (A1), the KL divergence can be expressed as

(A6) $$\begin{array}{cc} D_{\mathrm{KL}}\left(P,Q|x\right) & =\int_{-\infty }^{+\infty }P_{Y|X}\left(y|x\right)\ln\left(P_{Y|X}\left(y|x\right)\right)dy-\int_{-\infty }^{+\infty }P_{Y|X}\left(y|x\right)\ln\left(Q_{Y|X}\left(y|x\right)\right)dy\\ \; & =-h_{\mathrm{NCX}}\left(x\right)-E_{\mathrm{NCX}}\left[\ln\left(Q_{Y|X}\left(y|x\right)\right)\right], \end{array}$$

where $h_{\mathrm{NCX}}\left(x\right)$ denotes the differential entropy of the NCX distribution (A2) given parameter *x*, and $E_{\mathrm{NCX}}\left(\cdot \right)$ denotes the expectation over the NCX distribution (A2). The first term can be expressed as

(A7) $$\begin{array}{cc} h_{\mathrm{NCX}}\left(x\right) & =-E_{\mathrm{NCX}}\left[\ln\left(P_{Y|X}\left(y|x\right)\right)\right]\\ \; & =-E_{\mathrm{NCX}}\left[\ln\left(\frac{y^{2}}{x}\exp\left(-\frac{y^{2}+x^{2}}{2}\right)I_{1}\left(xy\right)\right)\right]\\ \; & =-2E_{\mathrm{NCX}}\left[\ln\left(y\right)\right]-E_{\mathrm{NCX}}\left[\ln\left(I_{1}\left(xy\right)\right)\right]+x^{2}+\ln\left(x\right)+2, \end{array}$$

while the second term can be written as

(A8) $$\begin{array}{cc} E_{\mathrm{NCX}}\left[\ln\left(Q_{Y|X}\left(y|x\right)\right)\right] & =E_{\mathrm{NCX}}\left[\ln\left(\frac{1}{\sqrt{2\pi \sigma_{\mathrm{NCX}}^{2}\left(x\right)}}\exp\left(-\frac{{\left(y-\mu_{NCX}\left(x\right)\right)}^{2}}{2\sigma_{\mathrm{NCX}}^{2}\left(x\right)}\right)\right)\right]\\ \; & =\ln\frac{1}{\sqrt{2\pi \sigma_{\mathrm{NCX}}^{2}\left(x\right)}}-\frac{1}{2}. \end{array}$$

Since function $f\left(y\right)=\ln(I_{1}\left(xy\right))$ where *x* is a given non-negative constant and function g(y)=ln(y) are concave functions [39], Jensen’s inequality is applied to obtain an upperbound on the KL divergence as

(A9) $$\begin{array}{cc} D_{\mathrm{KL}}\left(P,Q|x\right)\; & =2E_{\mathrm{NCX}}\left[\ln\left(y\right)\right]+E_{\mathrm{NCX}}\left[\ln\left(I_{1}\left(xy\right)\right)\right]-x^{2}-\ln\frac{x}{\sqrt{2\pi \sigma_{\mathrm{NCX}}^{2}\left(x\right)}}-\frac{3}{2}\\ \; & \leq \ln\frac{{\left(\mu_{NCX}\left(x\right)\right)}^{2}I_{1}\left(x\mu_{NCX}\left(x\right)\right)\sqrt{2\pi \sigma_{\mathrm{NCX}}^{2}\left(x\right)}}{xe^{x^{2}+3/2}}=D_{\mathrm{ub}}\left(x\right). \end{array}$$

Next, we find the limit of the upper bound of KL divergence $D_{\mathrm{ub}}\left(x\right)$ using the limits of mean $\mu_{\mathrm{NCX}}$ and variance $\sigma_{\mathrm{NCX}}^{2}\left(x\right)$ already calculated in (A4) and (A5), that is

(A10) $$\lim_{x\to +\infty }D_{\mathrm{ub}}\left(x\right)=\lim_{x\to +\infty }\ln\frac{\left(3+x^{2}\right)I_{1}\left(x\sqrt{3+x^{2}}\right)\sqrt{2\pi }}{xe^{x^{2}+3/2}}=0.$$

At last, using the non-negativity of KL divergence, we have $0\leq \lim_{x\to +\infty }D_{\mathrm{KL}}\leq \lim_{x\to +\infty }D_{\mathrm{ub}}=0$, i.e., $\lim_{x\to +\infty }D_{\mathrm{KL}}=0$. Therefore, we can conclude that the KL divergence between (A2) and (A3) goes to zero when *x* is sufficiently large and this concludes the proof. □

## Appendix B

**Proof of Theorem** **1.** In this proof, we show that the gap between the NCX channel capacity and the mismatch capacity of the NCX channel given the approximate AWGN channel as auxiliary channel tends to zero as $X_{\mathrm{lb}}\to \infty$. Consider that the input random variable $X\in \{0\cup \left[X_{\mathrm{lb}},X_{\mathrm{ub}}\right]\}$ is separated into zero and nonzero sets. Then the PDF of *X* can be written as

(A11) $$P_{X}\left(x\right)=p_{0}\delta \left(x\right)+\left(1-p_{0}\right)P_{\hat{X}}\left(x\right),$$

where δ(x) denotes the Dirac delta function, and $P_{\hat{X}}\left(x\right)$ denotes the PDF of the nonzero input $\hat{X}$. Similarly, the output random variable *Y* can also be separated in a similar manners as

(A12) $$P_{Y}\left(y\right)=p_{0}P_{Y|X}\left(y|0\right)+\left(1-p_{0}\right)P_{\hat{Y}}\left(y\right),$$

where the $P_{\hat{Y}}\left(y\right)=\int_{\hat{X}}P_{Y|X}P_{\hat{X}}\left(x\right)dx$ denotes the PDF of the output corresponding to the nonzero input. The MI between input *X* and output *Y* is then given as

(A13) $$I\left(X;Y\right)=h\left(Y\right)-h\left(Y|X\right)=\int_{X}\int_{Y}P_{X}\left(x\right)P_{Y|X}\left(y|x\right)\log_{2}\frac{1}{P_{Y}\left(y\right)}dydx-\int_{X}\int_{Y}P_{X}\left(x\right)P_{Y|X}\left(y|x\right)\log_{2}\frac{1}{P_{Y|X}}dydx.$$

Substituting Equation (A12) in the output differential entropy h(Y), it is then rewritten as

(A14) $$\begin{array}{cc} h(Y)= & \int_{X_{\mathrm{lb}}}^{X_{\mathrm{ub}}}\int_{X_{\mathrm{lb}}/2}^{+\infty }P_{X}\left(x\right)P_{Y|X}\left(y|x\right)\log_{2}\frac{1}{P_{Y}\left(y\right)}dydx+\int_{-\infty }^{X_{\mathrm{lb}}/2}p_{0}P_{Y|X}\left(y|0\right)\log_{2}\frac{1}{P_{Y}\left(y\right)}dy\\ = & \int_{X_{\mathrm{lb}}}^{X_{\mathrm{ub}}}\int_{-X_{\mathrm{lb}}/2}^{+\infty }P_{X}\left(x\right)P_{Y^{\prime }|X}\left(y^{\prime }|x\right)\log_{2}\frac{1}{p_{0}P_{Y^{\prime }|X}\left(y^{\prime }|0\right)+\left(1-p_{0}\right)P_{\hat{Y^{\prime }}}^{\prime }\left(y^{\prime }\right)}dy^{\prime }dx\\ \; & +\int_{-\infty }^{X_{\mathrm{lb}}/2}p_{0}P_{Y|X}\left(y|0\right)\log_{2}\frac{1}{p_{0}P_{Y|X}\left(y|0\right)+\left(1-p_{0}\right)P_{\hat{Y}}\left(y\right)}dy, \end{array}$$

where we changed the variable of integral in the first term of the last equality as $y^{\prime }=y-X_{\mathrm{lb}}$. Taking the Taylor expansion of the logarithmic functions inside the two integrals of the right hand side of (A14) at $y^{\prime }=0$ and y=0, respectively, we obtain

(A15) $$\begin{array}{cc} h(Y)= & \int_{X_{\mathrm{lb}}}^{X_{\mathrm{ub}}}\int_{-X_{\mathrm{lb}}/2}^{+\infty }P_{X}\left(x\right)P_{Y^{\prime }|X}\left(y^{\prime }|x\right)\left[\log_{2}\frac{1}{\left(1-p_{0}\right)P_{\hat{Y^{\prime }}}\left(y^{\prime }\right)}+\sum_{k=1}^{\infty }\frac{{\left(-1\right)}^{k}}{k}{\left(\frac{p_{0}P_{Y^{\prime }|X}\left(y^{\prime }|0\right)}{\left(1-p_{0}\right)P_{\hat{Y^{\prime }}}\left(y^{\prime }\right)}\right)}^{k}\right]dy^{\prime }dx\\ \; & +\int_{-\infty }^{X_{\mathrm{lb}}/2}P_{0}P_{Y|X}\left(y|0\right)\left[\log_{2}\frac{1}{p_{0}P_{Y|X}\left(y|0\right)}+\sum_{k=1}^{\infty }\frac{{\left(-1\right)}^{k}}{k}{\left(\frac{\left(1-p_{0}\right)P_{\hat{Y}}\left(y\right)}{p_{0}P_{Y|X}\left(y|0\right)}\right)}^{k}\right]dy\\ = & \int_{X_{\mathrm{lb}}}^{X_{\mathrm{ub}}}\int_{-X_{\mathrm{lb}}/2}^{+\infty }P_{X}\left(x\right)P_{Y^{\prime }|X}\left(y^{\prime }|x\right)\log_{2}\frac{1}{\left(1-p_{0}\right)P_{\hat{Y^{\prime }}}\left(y^{\prime }\right)}dy^{\prime }dx+\hat{\Delta }\\ \; & +\int_{-\infty }^{X_{\mathrm{lb}}/2}P_{0}P_{Y|X}\left(y|0\right)\log_{2}\frac{1}{p_{0}P_{Y|X}\left(y|0\right)}dy+\Delta_{0}, \end{array}$$

where the $\hat{\Delta }$ and $\Delta_{0}$ are higher order terms for nonzero and zero input, respectively. At $X_{\mathrm{lb}}\to \infty$, $\hat{\Delta }=E\left[\sum_{k=1}^{\infty }\frac{{\left(-1\right)}^{k}}{k}{\left[\frac{p_{0}P^{\prime }\left(y^{\prime }|0\right)}{\left(1-p_{0}\right)P_{\hat{Y^{\prime }}}\left(y^{\prime }\right)}\right]}^{k}\right]$ and $\Delta_{0}=E\left[\sum_{k=1}^{\infty }\frac{{\left(-1\right)}^{k}}{k}{\left[\frac{\left(1-p_{0}\right)P_{\hat{Y}}\left(y\right)}{p_{0}P_{Y|X}\left(y|0\right)}\right]}^{k}\right]$ can be written in the form of expectations. They will therefore vanish since, for NCX distribution, $\lim_{X_{\mathrm{lb}}\to \infty }P_{Y^{\prime }|X}\left(y^{\prime }\leq -X_{\mathrm{lb}}/2|x\geq X_{\mathrm{lb}}\right)=0$ and $\lim_{X_{\mathrm{lb}}\to \infty }P_{Y|X}\left(y\geq X_{\mathrm{lb}}/2|0\right)=0$. Inserting (A15) in (A13), the MI is given as

(A16) $$\begin{array}{cc} I(X;Y)= & \int_{X_{\mathrm{lb}}}^{-X_{\mathrm{ub}}}\int_{X_{\mathrm{lb}}/2}^{+\infty }P_{X}\left(x\right)P_{Y^{\prime }|X}\left(y^{\prime }|x\right)\log_{2}\frac{P_{Y^{\prime }|X}\left(y^{\prime }|x\right)}{\left(1-p_{0}\right)P_{\hat{Y^{\prime }}}^{\prime }\left(y^{\prime }\right)}dy^{\prime }dx+\hat{\Delta }\\ \; & +\int_{-\infty }^{X_{\mathrm{lb}}/2}P_{0}P_{Y|X}\left(y|0\right)\log_{2}\frac{1}{p_{0}}dy+\Delta_{0}. \end{array}$$

The mismatch capacity is a proven lower bound of the capacity. Assuming mismatch decoder design based on the Gaussian distribution, $Q_{Y|X}\left(y|x\right)$, the mismatch capacity $I_{\mathrm{LB}}$ is defined as

(A17) $$I_{\mathrm{LB}}=\int_{X}\int_{Y}P_{X}\left(x\right)P_{Y|X}\left(y|x\right)\log_{2}\frac{Q_{Y|X}\left(y|x\right)}{Q_{Y}\left(y\right)}dydx,$$

where the mismatch output distribution $Q_{Y}\left(y\right)$ can be written in similar manner as (A12) as

(A18) $$Q_{Y}\left(y\right)=p_{0}Q_{Y|X}\left(y|0\right)+\left(1-p_{0}\right)Q_{\hat{Y}}\left(y\right),$$

where the $Q_{\hat{Y}}\left(y\right)=\int_{\hat{X}}Q_{Y|X}\left(y|x\right)P_{\hat{X}}\left(x\right)dx$ denotes the PDF of the output corresponding to the nonzero input. The mismatch capacity at $X_{\mathrm{lb}}$ can be obtained via similar approach as before as

(A19) $$\begin{array}{cc} I_{\mathrm{LB}}\left(X;Y\right)= & \int_{X_{\mathrm{lb}}}^{X_{\mathrm{ub}}}\int_{-X_{\mathrm{lb}}/2}^{+\infty }P_{X}\left(x\right)P_{Y^{\prime }|X}\left(y^{\prime }|x\right)\left[\log_{2}\frac{Q_{Y^{\prime }|X}\left(y^{\prime }|x\right)}{\left(1-p_{0}\right)Q_{\hat{Y^{\prime }}}\left(y^{\prime }\right)}+\sum_{k=1}^{\infty }\frac{{\left(-1\right)}^{k}}{k}{\left(\frac{p_{0}Q_{Y^{\prime }|X}\left(y^{\prime }|0\right)}{\left(1-p_{0}\right)Q_{\hat{Y^{\prime }}}\left(y^{\prime }\right)}\right)}^{k}\right]dy^{\prime }dx\\ \; & +\int_{-\infty }^{X_{\mathrm{lb}}/2}P_{0}P^{\prime }\left(y^{\prime }|0\right)\left[\log_{2}\frac{Q_{Y|X}\left(y|0\right)}{p_{0}Q_{Y|X}\left(y|0\right)}+\sum_{k=1}^{\infty }\frac{{\left(-1\right)}^{k}}{k}{\left(\frac{\left(1-p_{0}\right)Q_{\hat{Y}}\left(y\right)}{p_{0}Q_{Y|X}\left(y|0\right)}\right)}^{k}\right]dy\\ = & \int_{X_{\mathrm{lb}}}^{X_{\mathrm{ub}}}\int_{-X_{\mathrm{lb}}/2}^{+\infty }P_{X}\left(x\right)P_{Y^{\prime }|X}\left(y^{\prime }|x\right)\log_{2}\frac{Q^{\prime }\left(y|x\right)}{\left(1-p_{0}\right)Q_{\hat{Y^{\prime }}}\left(y^{\prime }\right)}dy^{\prime }dx+\hat{\nabla }\\ \; & +\int_{-\infty }^{X_{\mathrm{lb}}/2}P_{0}P_{Y|X}\left(y|0\right)\log_{2}\frac{1}{p_{0}}dy+\nabla_{0}, \end{array}$$

where the $\hat{\nabla }$ and $\nabla_{0}$ are higher order terms of the Taylor expansion for nonzero and zero inputs. Similarly, at $X_{\mathrm{lb}}\to \infty$, $\hat{\nabla }=E\left[\sum_{k=1}^{\infty }\frac{{\left(-1\right)}^{k}}{k}{\left[\frac{p_{0}Q_{Y^{\prime }|X}\left(y^{\prime }|0\right)}{\left(1-p_{0}\right)Q_{\hat{Y^{\prime }}}\left(y^{\prime }\right)}\right]}^{k}\right]$ and $\nabla_{0}=E\left[\sum_{k=1}^{\infty }\frac{{\left(-1\right)}^{k}}{k}{\left[\frac{\left(1-p_{0}\right)Q_{\hat{Y}}\left(y\right)}{p_{0}Q_{Y|X}\left(y|0\right)}\right]}^{k}\right]$ can be written in the form of expectations. They will therefore vanish, for AWGN channel, since $\lim_{X_{\mathrm{lb}}\to \infty }Q_{Y^{\prime }|X}\left(y^{\prime }\leq -X_{\mathrm{lb}}/2|x\geq X_{\mathrm{lb}}\right)=0$ and $\lim_{X_{\mathrm{lb}}\to \infty }Q_{Y|X}\left(y\geq X_{\mathrm{lb}}/2|0\right)=0$ for the Gaussian distribution. The gap between the MI I(X;Y) and its lower bound $I_{\mathrm{LB}}\left(X;Y\right)$ is then defined as

(A20) $$\begin{array}{cc} I_{\mathrm{gap}}= & I\left(X;Y\right)-I_{\mathrm{LB}}\left(X;Y\right)\\ = & \int_{X_{\mathrm{lb}}}^{X_{\mathrm{ub}}}\int_{-X_{\mathrm{lb}}/2}^{+\infty }P_{X}\left(x\right)P_{Y^{\prime }|X}\left(y^{\prime }|x\right)\log_{2}\frac{P_{Y^{\prime }|X}\left(y^{\prime }|x\right)}{\left(1-p_{0}\right)P_{\hat{Y^{\prime }}}\left(y^{\prime }\right)}\frac{\left(1-p_{0}\right)Q_{\hat{Y^{\prime }}}^{\prime }\left(y^{\prime }\right)}{Q_{Y^{\prime }|X}\left(y^{\prime }|x\right)}dy^{\prime }dx+\hat{\Delta }-\hat{\nabla }+\Delta_{0}-\nabla_{0}\\ = & \hat{\Delta }-\hat{\nabla }+\Delta_{0}-\nabla_{0}+\int_{X_{\mathrm{lb}}}^{X_{\mathrm{ub}}}\int_{-X_{\mathrm{lb}}/2}^{+\infty }P_{X}\left(x\right)P_{Y^{\prime }|X}\left(y^{\prime }|x\right)\log_{2}\frac{P_{Y^{\prime }|X}\left(y^{\prime }|x\right)}{Q_{Y^{\prime }|X}\left(y^{\prime }|x\right)}dy^{\prime }dx\\ \; & -\int_{X_{\mathrm{lb}}}^{X_{\mathrm{ub}}}\int_{-X_{\mathrm{lb}}/2}^{+\infty }P_{X}\left(x\right)P_{Y^{\prime }|X}\left(y^{\prime }|x\right)\log_{2}\frac{P_{\hat{Y^{\prime }}}\left(y^{\prime }\right)}{Q_{\hat{Y^{\prime }}}\left(y^{\prime }\right)}dy^{\prime }dx. \end{array}$$

At $X_{\mathrm{lb}}\to \infty$, the vanishing terms $\hat{\Delta }$, $\hat{\nabla }$, $\Delta_{0}$ and $\nabla_{0}$ tends to 0, Hence, the limit of $I_{\mathrm{gap}}$ is given by

(A21) $$\begin{array}{cc} \lim_{X_{\mathrm{lb}}\to \infty }I_{\mathrm{gap}}= & \int_{X_{\mathrm{lb}}}^{X_{\mathrm{ub}}}P_{X}\left(x\right)\left(\int_{-\infty }^{+\infty }P_{Y^{\prime }|X}\left(y^{\prime }|x\right)\log_{2}\frac{P_{Y^{\prime }|X}\left(y^{\prime }|x\right)}{Q_{Y^{\prime }|X}\left(y^{\prime }|x\right)}dy\right)dx\\ \; & -\int_{-\infty }^{+\infty }\left(\int_{X_{\mathrm{lb}}}^{X_{\mathrm{ub}}}P_{X}\left(x\right)P_{Y^{\prime }|X}\left(y^{\prime }|x\right)dx\right)\log_{2}\frac{P_{\hat{Y^{\prime }}}^{\prime }\left(y^{\prime }\right)}{Q_{\hat{Y^{\prime }}}^{\prime }\left(y^{\prime }\right)}dy^{\prime }\\ \; & =\int_{X_{\mathrm{lb}}}^{X_{\mathrm{ub}}}P_{X}\left(x\right)D_{\mathrm{KL}}\left(P^{\prime },Q^{\prime }|x\right)dx-\left(1-p_{0}\right)D_{\mathrm{KL}}\left(P_{\hat{Y^{\prime }}}^{\prime },Q_{\hat{Y^{\prime }}}^{\prime }\right), \end{array}$$

where the second term in (A20) is a nonnegative KL divergence term, hence, the $I_{\mathrm{gap}}$ at $X_{\mathrm{lb}}\to \infty$ is bounded by

(A22) $$0\leq \lim_{X_{\mathrm{lb}}\to \infty }I_{\mathrm{gap}}\leq \int_{X_{\mathrm{lb}}}^{X_{\mathrm{ub}}}P_{X}\left(x\right)D_{\mathrm{KL}}\left(P^{\prime },Q^{\prime }|x\right)dx=\int_{X_{\mathrm{lb}}}^{X_{\mathrm{ub}}}P_{X}\left(x\right)D_{\mathrm{KL}}\left(P,Q|x\right)dx,$$

which is the expectation of the KL divergence over the nonzero range of *X*. According to Proposition 1, $\lim_{x\to \infty }D_{\mathrm{KL}}\left(P,Q|x\in \left[X_{\mathrm{lb}},X_{\mathrm{ub}}\right]\right)$ for NCX PDF $P_{Y|X}\left(y|x\right)$ and Gaussian PDF $Q_{Y|X}\left(y|x\right)$ tends to 0, therefore, the upper bound of the $I_{\mathrm{gap}}$ also tends to 0. This completes the proof. □

## Appendix C

**Proof of Proposition** **2.** Consider the input random variable $X\in \{0\cup \left[X_{\mathrm{lb}},X_{\mathrm{ub}}\right]\}$ is separated into zero and nonzero sets. Then the probability density function of *X* can be

(A23) $$P_{X}\left(x\right)=p_{0}\delta \left(x\right)+\left(1-p_{0}\right)P_{\hat{X}}\left(x\right),$$

where δ(x) denotes the Dirac delta function, and $P_{\hat{X}}\left(x\right)$ denotes the PDF of the nonzero input $\hat{X}$. Similarly, the output random variable *Y* can also be separated with similar manners as

(A24) $$P_{Y}\left(y\right)=p_{0}P_{Y|X}\left(y|0\right)+\left(1-p_{0}\right)P_{\hat{Y}}\left(y\right),$$

where the $P_{\hat{Y}}\left(y\right)=\int_{\hat{X}}P_{Y|X}\left(y|x\right)P_{\hat{X}}\left(x\right)dx$ denotes the PDF of the output corresponding to the nonzero input. Using the Taylor expansion as in Equation (A15), Appendix B, and considering the AWGN channel model, defined by $P_{Y|X}\left(y|x\right)$, the MI is given as

(A25) $$\begin{array}{cc} I(X;Y)= & h(Y)-h(Y|X)=h(Y)-h(\Gamma )\\ = & \int_{-\infty }^{+\infty }P_{Y}\left(y\right)\log_{2}\frac{1}{P_{Y}\left(y\right)}dy-\log_{2}\sqrt{2\pi e}\\ = & \int_{X_{\mathrm{lb}}}^{X_{\mathrm{ub}}}\int_{-X_{\mathrm{lb}}/2}^{+\infty }P_{X}\left(x\right)P_{Y^{\prime }|X}\left(y^{\prime }|x\right)\log_{2}\frac{1}{\left(1-p_{0}\right)P_{\hat{Y^{\prime }}}^{\prime }\left(y^{\prime }\right)}dy^{\prime }dx+\hat{\Delta }\\ \; & +\int_{-\infty }^{X_{\mathrm{lb}}/2}P_{0}P_{Y|X}\left(y|0\right)\log_{2}\frac{1}{p_{0}P_{Y|X}\left(y|0\right)}dy+\Delta_{0}-\log_{2}\sqrt{2\pi e}, \end{array}$$

where we changed the variable of integral in the first term of the last equality as $y^{\prime }=y-X_{\mathrm{lb}}$. At $X_{\mathrm{lb}}\to \infty$, $\hat{\Delta }=E_{\hat{X}\times Y^{\prime }}\left[\sum_{k=1}^{\infty }\frac{{\left(-1\right)}^{k}}{k}{\left[\frac{p_{0}P^{\prime }\left(y^{\prime }|0\right)}{\left(1-p_{0}\right)P_{\hat{Y^{\prime }}}\left(y^{\prime }\right)}\right]}^{k}\right]$ and $\Delta_{0}=E_{X_{0}\times Y}\left[\sum_{k=1}^{\infty }\frac{{\left(-1\right)}^{k}}{k}{\left[\frac{\left(1-p_{0}\right)P_{\hat{Y}}\left(y\right)}{p_{0}P_{Y|X}\left(y|0\right)}\right]}^{k}\right]$ can be written in the form of expectations. They will therefore vanish since, for AWGN channel, $\lim_{X_{\mathrm{lb}}\to \infty }P_{Y^{\prime }|X}\left(y^{\prime }\leq X_{\mathrm{lb}}/2|x\;\geq \;X_{\mathrm{lb}}\right)=0$ and $\lim_{X_{\mathrm{lb}}\to \infty }P_{Y|X}\left(y\geq X_{\mathrm{lb}}/2|0\right)=0$. Hence, we will have

(A26) $$\begin{array}{cc} I_{\infty }\left(X;Y\right)= & \lim_{X_{\mathrm{lb}\to \infty }}I\left(X;Y\right)\\ = & \int_{X_{\mathrm{lb}}}^{X_{\mathrm{ub}}}\int_{-\infty }^{+\infty }P_{X}\left(x\right)P_{Y|X}\left(y|x\right)\log_{2}\frac{1}{\left(1-p_{0}\right)P_{\hat{Y}}\left(y\right)}dydx\\ \; & +\int_{-\infty }^{+\infty }P_{0}P_{Y|X}\left(y|0\right)\log_{2}\frac{1}{p_{0}P_{Y|X}\left(y|0\right)}dy-\log_{2}\sqrt{2\pi e},\\ = & p_{0}\log_{2}\frac{1}{p_{0}}+\left(1-p_{0}\right)\log_{2}\frac{1}{1-p_{0}}+\left(1-p_{0}\right)\left(h\left(\hat{Y}\right)-\log_{2}\sqrt{2\pi e}\right)\\ = & h_{0}+\left(1-p_{0}\right)I\left(\hat{X};\hat{Y}\right), \end{array}$$

where $h_{0}=p_{0}\log_{2}\frac{1}{p_{0}}+\left(1-p_{0}\right)\log_{2}\frac{1}{1-p_{0}}$, and $I(\hat{X};\hat{Y})$ denotes the MI between the nonzero input $\hat{X}$ and its corresponding output $\hat{Y}$. The time- scaled capacity formulation in (19) is then given as

(A27) $$\begin{array}{cc} C= & \underset{P_{X}\left(x\right):X\in \left\{0\right\}\cup \left[X_{\mathrm{lb}},X_{\mathrm{ub}}\right]}{\sup}R_{\infty }\left(X;Y\right)=\underset{P_{X}\left(x\right):X\in \left\{0\right\}\cup \left[X_{\mathrm{lb}},X_{\mathrm{ub}}\right]}{\sup}\frac{I_{\infty }}{t_{s}\left(A_{\min},\delta \right)}\\ = & \underset{p_{0},P_{\hat{X}}\left(x\right):x\in \left[X_{\min},X_{\mathrm{ub}}\right]}{\sup}\frac{\sigma_{N}^{2}X_{\min}^{2}}{2\ln(2/\delta -1)}\left(h_{0}+\left(1-p_{0}\right)I\left(\hat{X};\hat{Y}\right)\right). \end{array}$$

Let $P_{X}^{*}\left(x\right)$ denote the capacity achieving distribution for the problem in (A27), which is fully defined by $p_{0}^{*}$, $X_{\min}^{*}$ and the PDF of nonzero input, $P_{\hat{X}}^{*}$. Next, we will show that this distribution is discrete with a finite number of mass points, which in turn implies the statement of this proposition. We first show that the capacity achieving distribution $P_{\hat{X}}^{*}$ is also the solution of the following optimization problem

(A28) $$\underset{P_{\hat{X}}\left(x\right):x\in \left[X_{\min}^{*},X_{\mathrm{ub}}\right]}{\sup}I\left(\hat{X};\hat{Y}\right).$$

Let $P_{\hat{X}}^{\circ }$ be any arbitrary distribution within the feasible set of the problem in (A28), implying that $X_{\min}^{\circ }\geq X_{\min}^{*}$. Since $P_{\hat{X}}^{*}$ defines the capacity achieving distribution for the problem in (A27), it yields a time-scaled MI larger than that of any arbitrary distribution such as $P_{\hat{X}}^{\circ }$. Therefore we can write

(A29) $$\begin{array}{cc} \frac{\sigma_{N}^{2}{X_{\min}^{*}}^{2}}{2\ln(2/\delta -1)}\left(h_{0}{|}_{p_{0}^{*}}+\left(1-p_{0}^{*}\right)I^{*}\left(\hat{X};\hat{Y}\right)\right) & \geq \frac{\sigma_{N}^{2}{X_{\min}^{\circ }}^{2}}{2\ln(2/\delta -1)}\left(h_{0}{|}_{p_{0}^{*}}+\left(1-p_{0}^{*}\right)I^{\circ }\left(\hat{X};\hat{Y}\right)\right), \end{array}$$

where $I^{*}\left(\hat{X};\hat{Y}\right)$ and $I^{\circ }\left(\hat{X};\hat{Y}\right)$ are the MI given by $P_{\hat{X}}^{*}$ and $P_{\hat{X}}^{\circ }$, respectively. Simplifying (A29) and using the fact that $X_{\min}^{\circ }\geq X_{\min}^{*}$, we have

(A30) $$\begin{array}{cc} h_{0}{|}_{p_{0}^{*}}+\left(1-p_{0}^{*}\right)I^{*}\left(\hat{X};\hat{Y}\right) & \geq \frac{{X_{\min}^{\circ }}^{2}}{{X_{\min}^{*}}^{2}}\left(h_{0}{|}_{p_{0}^{*}}+\left(1-p_{0}^{*}\right)I^{\circ }\left(\hat{X};\hat{Y}\right)\right)\geq h_{0}{|}_{p_{0}^{*}}+\left(1-p_{0}^{*}\right)I^{\circ }\left(\hat{X};\hat{Y}\right), \end{array}$$

which implies that $I^{*}\left(\hat{X};\hat{Y}\right)\geq I^{\circ }\left(\hat{X};\hat{Y}\right)$. Since this is true for any arbitrary $P_{\hat{X}}^{\circ }$ within the feasible set of the problem in (A28), we can conclude that $P_{\hat{X}}^{*}$ is also the optimal distribution for the problem in (A28). Note that the problem in (A28) is equivalent to the amplitude constrained AWGN channel capacity problem presented in [34]. In [34], Smith proved that the capacity achieving distribution for such channels are discrete with a finite number of mass points. Thus, the optimal $P_{\hat{X}}^{*}$ and thereby $P_{X}^{*}$ should be discrete with a finite number of mass points as well, which concludes this proof. □

## Appendix D

**Proof of Theorem** **2.** Recalling the approximate time-scaled MI function is given as

(A31) $$\begin{array}{cc} R_{\mathrm{app}}\left(X;Y\right)= & \frac{\sigma_{N}^{2}X_{\min}^{2}}{2\ln(2/\delta -1)}\left[p_{0}\log_{2}\frac{1}{p_{0}}+\left(1-p_{0}\right)\log_{2}\frac{X_{\max}-X_{\min}}{1-p_{0}}-\left(1-p_{0}\right)\log_{2}\left(\sqrt{2\pi e}\right)\right]. \end{array}$$

In order to find the maximum of the function (A31) analytically, its first order partial derivatives with respect to $p_{0}$, $X_{\min}$ and $X_{\max}$, are first derived. These first order partial derivatives are given as

(A32) $$\begin{array}{cc} \frac{\partial R_{\mathrm{app}}}{\partial X_{\max}}= & \frac{\sigma_{N}^{2}X_{\min}^{2}}{2\ln(2/\delta +1)}\frac{1-p_{0}}{X_{\max}-X_{\min}},\\ \frac{\partial R_{\mathrm{app}}}{\partial X_{\min}}= & \frac{2\sigma_{N}^{2}X_{\min}}{2\ln(2/\delta +1)}\left(p_{0}\ln\frac{1}{p_{0}}+\left(1-p_{0}\right)\ln\frac{X_{\max}-X_{\min}}{1-p_{0}}-\left(1-p_{0}\right)\ln\sqrt{2\pi e}\right)- \end{array}$$

(A33) $$\begin{array}{cc} \; & \frac{\sigma_{N}^{2}X_{\min}^{2}}{2\ln(2/\delta +1)}\left(\frac{1-p_{0}}{X_{\max}-X_{\min}}\right), \end{array}$$

(A34) $$\begin{array}{cc} \frac{\partial R_{\mathrm{app}}}{\partial p_{0}}= & \frac{\sigma_{N}^{2}X_{\min}^{2}}{2\ln(2/\delta +1)}\left(\ln\frac{1}{p_{0}}-\ln\frac{X_{\max}-X_{\min}}{1-p_{0}}+\ln\sqrt{2\pi e}\right), \end{array}$$

Notice that (A32) is positive because $X_{\max}>X_{\min}$ and $p_{0}<1$. This implies that the approximate time-scaled MI, $R_{\mathrm{app}}\left(\cdot \right)$, monotonically increases with respect to $X_{\max}$, thus, $R_{\mathrm{app}}\left(\cdot \right)$ maximizes at the boundary as

(A35) $$X_{\max}^{*}=X_{\mathrm{ub}}.$$

Now setting the partial derivative in (A33) to zero and using the boundary condition above, we obtain the following nonlinear equation that needs to be solved to obtain the possible optimal value of $X_{\min}$ denoted by $X_{\min}^{*}$.

(A36) $$2\ln\frac{X_{\mathrm{ub}}-X_{\min}^{*}+\sqrt{2\pi e}}{\sqrt{2\pi e}}=\frac{X_{\min}^{*}}{X_{\mathrm{ub}}-X_{\min}^{*}+\sqrt{2\pi e}}.$$

Note that the solution to this nonlinear equation can be written based on the Lambert W function W(·) as

(A37) $$X_{\min}^{*}=\left(X_{\mathrm{ub}}+\sqrt{2\pi e}\right)\left[1-\frac{1}{2W\left(\frac{X_{\mathrm{ub}}}{2\sqrt{2\pi }}+\frac{\sqrt{e}}{2}\right)}\right].$$

Then, the corresponding probability of the zero mass point can be derived by setting (A34) to zero and using the results above as

(A38) $$p_{0}^{*}=\frac{\sqrt{2\pi e}}{X_{\mathrm{ub}}-X_{\min}^{*}+\sqrt{2\pi e}}=\frac{2\sqrt{2\pi e}W\left(\frac{X_{\mathrm{ub}}}{2\sqrt{2\pi }}+\frac{\sqrt{e}}{2}\right)}{X_{\mathrm{ub}}+\sqrt{2\pi e}}.$$

Now, in order to show the optimally of $p_{0}^{*}$ and $X_{\min}^{*}$ derived above, the second order partial derivative test should be performed. The second order partial derivative with respect to $p_{0}$ and $X_{\min}$ is taken first and are given as

(A39) $$\frac{\partial^{2}R_{\mathrm{app}}}{\partial p_{0}^{2}}=-\frac{\sigma_{N}^{2}X_{\min}^{2}}{2\ln(2/\delta +1)}\left(\frac{1}{p_{0}}+\frac{1}{1-p_{0}}\right),$$

(A40) $$\begin{array}{cc} \frac{\partial^{2}R_{\mathrm{app}}}{\partial X_{\min}^{2}}=\frac{\sigma_{N}^{2}}{2\ln(2/\delta +1)}[ & 2(p_{0}\ln\frac{1}{p_{0}}+\left(1-p_{0}\right)\ln\frac{X_{\mathrm{ub}}-X_{\min}}{1-p_{0}}-\left(1-p_{0}\right)\ln\sqrt{2\pi e})-\\ \; & 4X_{\min}\frac{1-p_{0}}{X_{\mathrm{ub}}-X_{\min}}-X_{\min}^{2}\frac{1-p_{0}}{{\left(X_{\mathrm{ub}}-X_{\min}\right)}^{2}}]. \end{array}$$

The mixed second order partial derivatives are also required, which are given as

(A41) $$\frac{\partial^{2}R_{\mathrm{app}}}{\partial p_{0}\partial X_{\min}}=\frac{\partial^{2}R_{\mathrm{app}}}{\partial X_{\min}\partial p_{0}}=\frac{\sigma_{N}^{2}}{2\ln(2/\delta +1)}\left[2X_{\min}\ln\frac{\sqrt{2\pi e}\left(1-p_{0}\right)}{p_{0}\left(X_{\mathrm{ub}}-X_{\min}\right)}+\frac{X_{\min}^{2}}{X_{\mathrm{ub}}-X_{\min}}\right].$$

By inspecting Equations (A39) and (A40), one may find that the second order partial derivatives are less than zero at $p_{0}=p_{0}^{*}$ and $X_{\min}=X_{\min}^{*}$, while the determinant of the Hessian matrix, $\frac{\partial^{2}R_{\mathrm{app}}}{\partial p_{0}^{2}}\frac{\partial^{2}R_{\mathrm{app}}}{\partial X_{\min}^{2}}-\frac{\partial^{2}R_{\mathrm{app}}}{\partial p_{0}\partial X_{\min}}\frac{\partial^{2}R_{\mathrm{app}}}{\partial X_{\min}\partial p_{0}}$, is larger than zero. Hence, the maximum of the time-scaled MI in (A31) is obtained at the optimal points $X_{\max}^{*}=X_{\mathrm{ub}}$, $p_{0}^{*}$ and $X_{\min}^{*}$ defined above as

(A42) $$C_{\mathrm{app}}=R_{\mathrm{app}}{\left(X;Y\right)|}_{p_{0}^{*},X_{\min}^{*},X_{\max}^{*}}.$$

 □
